# Decursin Alleviates Mechanical Allodynia in a Paclitaxel-Induced Neuropathic Pain Mouse Model

**DOI:** 10.3390/cells10030547

**Published:** 2021-03-04

**Authors:** Dang Bao Son, Woosik Choi, Mingu Kim, Eun Jin Go, Dabeen Jeong, Chul-Kyu Park, Yong Ho Kim, Hanki Lee, Joo-Won Suh

**Affiliations:** 1 Center for Nutraceutical and Pharmaceutical Materials, Myongji University, Yongin 17058, Korea; baoson.dang@gmail.com (D.B.S.); woos5051@gmail.com (W.C.); jdabin16@gmail.com (D.J.); 2 Gachon Pain Center and Department of Physiology, College of Medicine, Gachon University, Incheon 21999, Korea; nkmglife@naver.com (M.K.); navy2474@gachon.ac.kr (E.J.G.); pck0708@gachon.ac.kr (C.-K.P.); 3 Department of Health Sciences and Technology, GAIHST, Gachon University, Incheon 21999, Korea

**Keywords:** CINP, decursin, recovery of damaged neuronal network, TRPV1 antagonist, lead compound

## Abstract

Chemotherapy-induced neuropathic pain (CINP) is a severe adverse effect of platinum- and taxane-derived anticancer drugs. The pathophysiology of CINP includes damage to neuronal networks and dysregulation of signal transduction due to abnormal Ca^2+^ levels. Therefore, methods that aid the recovery of neuronal networks could represent a potential treatment for CINP. We developed a mouse model of paclitaxel-induced peripheral neuropathy, representing CINP, to examine whether intrathecal injection of decursin could be effective in treating CINP. We found that decursin reduced capsaicin-induced intracellular Ca^2+^ levels in F11 cells and stimulated neurite outgrowth in a concentration-dependent manner. Decursin directly reduced mechanical allodynia, and this improvement was even greater with a higher frequency of injections. Subsequently, we investigated whether decursin interacts with the transient receptor potential vanilloid 1 (TRPV1). The web server SwissTargetPrediction predicted that TRPV1 is one of the target proteins that may enable the effective treatment of CINP. Furthermore, we discovered that decursin acts as a TRPV1 antagonist. Therefore, we demonstrated that decursin may be an important compound for the treatment of paclitaxel-induced neuropathic pain that functions via TRPV1 inhibition and recovery of damaged neuronal networks.

## 1. Introduction

Chemotherapy-induced neuropathic pain (CINP) is a severe side-effect of platinum and taxane-derived anti-cancer drugs. The incidence of CINP increases with an increase in the frequency and dosage of anticancer agents [1]. The principal symptoms of CINP include paresthesia and dysesthesia, which are induced by touch and warm or cool temperatures [1]. In severe cases, CINP can lead to complete impairment of mobility and severe disability [2]. Moreover, structural brain changes, such as axonopathy, small-fiber degeneration, demyelination, and atrophy, are often observed in the peripheral nerves of patients with CINP and rodent models of CINP; these changes are primarily caused by neurotoxicity induced by anticancer agents [2]. Currently, there is a remarkable lack of US Food and Drug Administration-approved drugs for CINP, despite the severity of this condition [3].

Decursin, which is one of the active compounds present in *Angelica gigas* Nakai, a Korean medicinal herb, is known to have several biological actions, including anti-inflammatory, wound healing, and anticancer effects [4,5,6]. In a previous study, we found that decursin could upregulate the transcription of genes encoding extracellular matrix remodeling proteins, inflammatory cytokines, and growth factors in human keratinocytes [7]. Interestingly, the genes upregulated by decursin are closely related to the maintenance of neuronal networks and recovery of damaged neuronal networks [7]. Moreover, it has been reported that CINP can cause severe damage to the peripheral nervous system [1]. Therefore, we hypothesized that decursin could be effective in ameliorating CINP. To this end, we investigated the analgesic activity of decursin in in vitro and in vivo experiments and examined its mechanism of action in reducing CINP.

## 2. Materials and Methods

### 2.1. Monitoring Chemical-Induced Intracellular Calcium Ion Levels in F11 Cells

In this experiment, we used F11 cells instead of dorsal root ganglion cells to avoid some typical drawbacks. Namely, the use of dorsal root ganglion cells has been associated with the need to sacrifice a large number of animals, labor-intensive isolation procedures, heterogeneity of the cytochemical phenotype, and the presence of non-neuronal cells that can cause artifacts [8]. F11 cells were grown in a culture dish (100 mm diameter, SPL Life Science, Pocheon, Korea) to 90% confluency at 37 °C. The cells (1 × 10^5^ cells/mL) were then transferred to black 96-well optical-bottom plates with a glass coverslip (Thermo Fisher Scientific, Waltham, MA, USA) and incubated at 37 °C for 24 h for cell attachment. After incubation, the medium was discarded and the wells were washed thrice with 200 μL of buffer (pH 7.4), which was composed of 145 mM NaCl (Sigma-Aldrich, Milwaukee, WI, USA), 5 mM KCl (Sigma-Aldrich, Milwaukee, WI, USA), 1 mM CaCl_2_ (Sigma-Aldrich, Milwaukee, WI, USA), 1 mM MgCl_2_ (Sigma-Aldrich, Milwaukee, WI, USA), 10 mM 4-(2-hydroxyethyl)-1-piperazineethanesulfonic acid (HEPES, Sigma-Aldrich, Milwaukee, WI, USA), and 10 mM glucose. Subsequently, 100 μL of the buffer, containing 5 μM of Fura-2 AM (ThermoFisher Scientific, Waltham, MA, USA) and 0.1% of pluronic F-127 (ThermoFisher Scientific, Waltham, MA, USA), was added to each well and incubated at 37 °C for 30 min. A multi-well plate reader (Tecan Infinite M200, Tecan, Männedorf, Switzerland) was set up below to monitor intracellular Ca^2+^ levels in F11 cells. The emission fluorescence intensity at 510 nm for each individual well was determined by alternating excitation wavelengths of 340 nm and 380 nm for 3 s each for 30 cycles. After 9 cycles, 500 nM of capsaicin (Sigma-Aldrich, Milwaukee, WI, USA) dissolved in dimethyl sulfoxide (DMSO, Sigma-Aldrich, Milwaukee, WI, USA) and decursin (each 0.5 μL, Sigma-Aldrich, Milwaukee, WI, USA) with capsaicin were added to the control and treatment groups, respectively. In the case of menthol (Sigma-Aldrich, Milwaukee, WI, USA), we treated with 200 μM of menthol dissolved in deionized water instead of capsaicin. We used the average emission fluorescence intensity at 510 nm during the first 9 cycles as the baseline and divided the emission fluorescence intensities of each cycle by the baseline to normalize the data. Finally, we used the average of the data corresponding to 10 cycles after the 10th cycle to calculate the normalized Ca^2+^ level in each group.

### 2.2. Measurement of Neurite Outgrowth of F11 Cells in the Presence of Paclitaxel

F11 cells were grown in a culture dish (100 mm diameter) to 90% confluency at 37 °C. The cells were transferred to a polymer coverslip-bottomed culture dish (35 mm diameter, ibidi, Gräfelfing, Germany) and incubated for 12 h in 10 nM of paclitaxel dissolved in DMSO at 37 °C. After incubation and washing with medium, the cells were treated with different concentrations of decursin dissolved in DMSO with fresh medium and incubated at 37 °C for 12 h. The Neurite Outgrowth Staining Kit (Invitrogen, Waltham, MA, USA) was used to visualize neurite outgrowth, according to the instruction manual. The medium was discarded after incubating the F11 cells with decursin at 37 °C for 12 h, and the culture dish was washed twice with Hank’s Balanced Salt Solution, containing calcium and magnesium (Sigma-Aldrich, Milwaukee, WI, USA). A working solution containing 1× cell membrane stain and 1× cell viability indicator was subsequently added to the plate, and the plate was incubated at 37 °C for 15 min. The working solution was discarded and 1× Background Suppression dye solution was added to the plate. The cell membrane and cell viability were observed using the Cy3 (Chroma, Pauling Foothill Ranch, CA, USA) and FITC filter set (Chroma, Pauling Foothill Ranch, CA, USA), respectively. Subsequently, 10 images were captured per plate using a cooled charge-coupled device camera (Optinity, Korea Lab Tech., Seongnam, Korea) under an inverted fluorescence microscope equipped with a 20× objective lens (IX73, Olympus, Tokyo, Japan). ImageJ software (1.53, National Institutes of Health, Betheda, MD, USA) was used to analyze neurite number and length.

### 2.3. Establishment of the Mouse Model of Paclitaxel-Induced Neuropathic Pain

The experimental protocol (GU-2018-02) for the management and care of animals was reviewed and approved by the Animal Care and Use Committee of Gachon University, Incheon, South Korea.

Eight-week-old adult C57BL/6J male and female mice were purchased from OrientBio, South Korea, and acclimated to the laboratory for 1 week. These mice were allowed free access to a general mouse diet and tap water in their cage environment. The cage room was maintained at 22 ± 2 °C with a 12-h light/dark cycle. Paclitaxel (Sigma-Aldrich, Milwaukee, WI, USA) was dissolved in DMSO and diluted with a mixture of ethanol (Sigma-Aldrich, Milwaukee, WI, USA) and Cremophor EL (Sigma-Aldrich, Milwaukee, WI, USA) at a 1:1 ratio. Paclitaxel (2 mg/kg, diluted with phosphate-buffered saline) was injected intraperitoneally four times at three-day intervals, over 12 days, to establish the paclitaxel-induced neuropathic model. The mechanical allodynia test was conducted on days 7 and 14 after the first injection of paclitaxel, and we selected mice that exhibited a 50% withdrawal threshold (g) that was below 0.3 to investigate the effect of decursin.

### 2.4. Determination of the Effect of Decursin in the Mouse Model of Paclitaxel-Induced Neuropathic Pain

For this experiment, decursin was first dissolved in DMSO, and this solution was diluted in Dulbecco’s phosphate-buffered saline by 10-fold. Each mouse was intrathecally injected with 5 μL of diluted decursin three times at 2-day intervals, for 6 days, to examine whether decursin could improve mechanical allodynia in the mouse model of paclitaxel-induced neuropathic pain. Intrathecal injections into the L5-L6 intervertebral space of unanesthetized mice were made using a 10 µL Hamilton syringe connected to a 30-gauge needle [9]. The effect of this compound was measured using the 50% paw withdrawal threshold, based on the von Frey method [10]. The mice were habituated to an elevated platform with a mesh floor for 30 min. The plantar side of the hind paw was stimulated with calibrated von Frey filaments (NC12775-99, North Coast Medical, Morgan Hill, CA, USA). The 50% paw withdrawal thresholds were calculated using the up-down method [10]. The mechanical allodynia test for monitoring the analgesic activity of this compound was conducted every 2 days at an appropriate time after the injection of decursin, for a total of three times over 6 days.

### 2.5. Prediction of Target Proteins Using Swisstargetprediction

SwissTargetPrediction (http://www.swisstargetprediction.ch (accessed on 25 February 2021)) is a web tool that can be used to predict the most probable protein targets of small molecules, and which allows the user to select the host from among *Homo sapiens*, *Mus musculus*, and *Rattus norvergicus* [11]. We input the structure of decursin using the canonical Simplified Molecular Input Line Entry System string CC(=CC(=O)OC1CC2=C(C=C3C(=C2)C=CC(=O)O3)OC1(C)C)C, which was provided by PubChem (https://pubchem.ncbi.nlm.nih.gov (accessed on 25 February 2021)), into the SwissTargetPrediction web tool and selected protein targets that were closely associated with capsaicin-related proteins for *Homo sapiens*.

### 2.6. Monitoring Transient Receptor Potential Vanilloid 1 (TRPV1)-Mediated Ca^2+^ in Human TRPV1 Stable Cell Line-HEK293

We monitored TRPV1-mediated Ca^2+^ in the human TRPV1 stable cell line HEK293, based on the method described by Roh et al. [12]. Human TRPV1 HEK293 stable cells were placed on poly-D-lysine-coated coverslips (Sigma-Aldrich, Milwaukee, WI, USA) and loaded with 2 μM Fura-2 AM (Thermo Fisher Scientific, Waltham, MA, USA) for 40 min at 37 °C in Dulbecco’s Modified Eagle’s Medium (DMEM, Thermo Fisher Scientific, Waltham, MA, USA) and transferred to the chamber. Subsequently, they were placed onto an inverted microscope (BX51WI, Olympus, Tokyo, Japan) and perfused continuously with a balanced bath solution containing 140 mM NaCl, 5 mM KCl, 2 mM CaCl_2_, 1 mM MgCl_2_, 10 mM HEPES, and 10 mM glucose. The solution was adjusted to a pH of 7.4 using NaOH. Calcium imaging was performed at 25 °C. Cells were illuminated with a 175-W Xenon arc lamp, and excitation wavelengths (340/380 nm) were selected using a Lambda DG-4 monochromator wavelength changer (Shutter Instrument, Novato, CA, USA). The 340/480 nm fluorescence ratio was measured using digital video microfluorometry with an intensified camera (optiMOS, QImaging, Surrey, BC, USA) coupled to a microscope and software (Slidebook 6, 3i, Intelligent Imaging Innovations, Denver, CO, USA). The perfusion system was driven by gravity and had a flow speed of 1 mL/min. The cells were treated with this compound at different concentrations to determine the effect of decursin on capsaicin-induced TRPV1 activation. The half maximal inhibitory concentration (IC_50_) of decursin was calculated after dose–response fitting using OriginPro software (9.0, OriginLab, Northampton, MA, USA).

### 2.7. Immunoblotting

F11 cells were grown in a culture dish (100 mm diameter) to 90% confluency. After washing, the F11 cells were starved in DMEM with 0.5% fetal bovine serum for 12 h. After removing the starved medium, DMEM-treated F11 cells were treated with 2 μM of decursin with 10% fetal bovine serum as a growth medium. These cells were then harvested after incubation for 30 min. Cell lysates were prepared with RIPA buffer (Sigma-Aldrich, Milwaukee, WI, USA) containing kinase inhibitor cocktail (Sigma-Aldrich, Milwaukee, WI, USA)and protease inhibitor cocktail (Sigma-Aldrich, Milwaukee, WI, USA). After centrifugation with 12,000× *g* for 10 min, the soluble fractions were used for Western blotting.

The soluble fractions were separated using SDS-PAGE (30 μg per lane) and transferred onto a polyvinylidene difluoride membrane (PVDF, Sigma-Aldrich, Milwaukee, WI, USA). After blocking the membrane with TBS-T (150 mM NaCl, 10 mM Tric-HCL, 0.1% Tween 20, pH 8.0) containing 3% BSA (Sigma-Aldrich, Milwaukee, WI, USA) for 1 h, the membrane was incubated with primary antibodies against p-CREB (1:200, Santa Crux Biotechnology, Dallas, TX, USA), total-CREB (1:1000; Santa Cruz Biotechnology, Dallas, TX, USA), p-Akt (1:250; Cell Signaling, Danver, MA, USA), total-Akt (1:1000; Cell Signaling, Danver, MA, USA), and β-actin (1:2000; Millipore, Schwalbach, Germany) at 4 °C overnight diluted in TBS-T containing 0.5% BSA. This membrane was washed three times in TBS-T and incubated with Anti-Rabbit IgG-HRP (1: 5000; Calbiochem, San Diego, CA, USA) as a secondary antibody at room temperature for 2 h. After washing the membrane with TBS-T three times, each protein was detected by treatment of 1-Step Chloronaphthol Substrate Solution (Thermo Fisher Scientific, Waltham, MA, USA). After scanning each membrane, the quantification of each protein band was analyzed using ImageJ (National Institutes of Health, Bethesda, MD, USA).

### 2.8. Statistical Analysis

Data were analyzed using Student’s *t*-tests and paired *t*-tests. A *p*-value of <0.05 and <0.01 was considered as statistically significant. The computer program Excel (Microsoft 365, Microsoft, Redmond, WA, USA) was used in all statistical analyses. All data are expressed as the mean ± SEM.

## 3. Results

### 3.1. Decursin Reduced the Sudden Increase of Intracellular Ca^2+^ in F11 Cells

**Figure 1:**
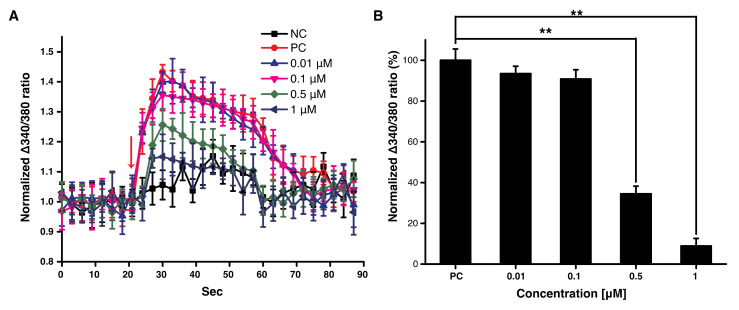
Intracellular Ca^2+^ levels after decursin treatment in F11 cells in the presence of capsaicin. (**A**) Intracellular Ca^2+^ levels after treatment with decursin every 3 s. The red arrow indicates the duration of treatment with capsaicin and decursin. NC: The negative control treated with DMSO only; PC: The positive control treated with 500 nM of capsaicin, without decursin. (**B**) Average intracellular Ca^2+^ levels after treatment with decursin. The average was quantified from the normalized Δ340/380 ratio for 10 cycles after treatment with the decursin solution at the 10th cycle, as shown in (A). The normalized Δ340/380 ratio was calculated using the following formula: [ratio of fluorescence intensity at 510 nm (emission) to that at 340 nm (excitation)]/[ratio of fluorescence intensity at 510 nm (emission) to that at 380 nm (excitation)]. ** *p* < 0.01, assessed using paired *t*-tests. This experiment was conducted in triplicate.

We treated F11 cells with different concentrations of decursin in the presence of capsaicin to determine whether decursin could reduce intracellular Ca^2+^ levels in damaged neuronal cells. Previous work has reported that capsaicin induces typical pain symptoms such as allodynia and hyperalgesia in vivo [13]. Thus, we treated F11 cells with capsaicin to examine intracellular Ca^2+^ level. The intracellular levels of Ca^2+^ increased dramatically following treatment with capsaicin, and slowly reduced to the basal level within 70 s (Figure 1A) in the positive-control group. However, the rapid elevation in intracellular Ca^2+^ levels was inhibited in a concentration-dependent manner in the decursin-treated group (Figure 1A). Treatment with 0.5 and 1 μM decursin significantly inhibited the elevation of intracellular Ca^2+^ by up to 64.5% and 91.2%, respectively (Figure 1B). In addition, we carried out this experiment using menthol (instead of capsaicin), which also increases intracellular Ca^2+^ in damaged neuronal cells. Interestingly, decursin did not effectively reduce intracellular Ca^2+^ increases that were induced by menthol treatment (Supplementary Figure S1). This implied that decursin might contribute to a decrease in capsaicin-induced membrane depolarization, which is principally observed in neurons involved in CINP owing to the higher levels of intracellular Ca^2+^ [1].

### 3.2. Decursin Induced Neuronal Differentiation of F11 Cells by Increasing Neurite Outgrowth

**Figure 2:**
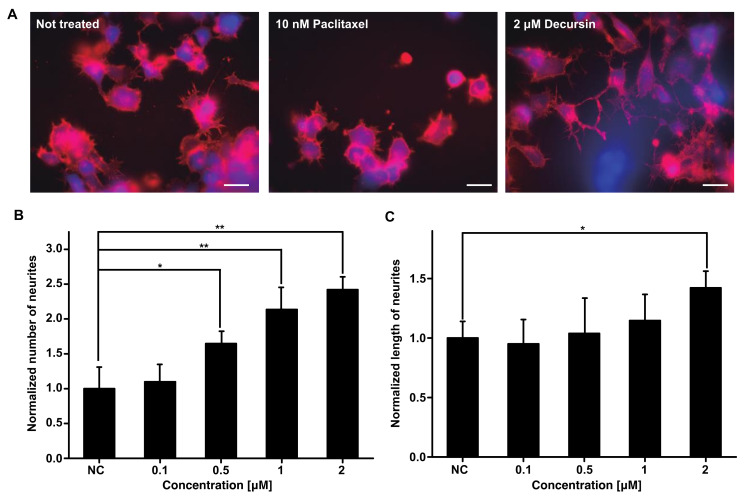
Number and length of F11 neurites in the presence of paclitaxel by decursin treatment. (**A**) The left, middle, and right images represent non-treatment, treatment with 10 nM of paclitaxel, and decursin treatment, respectively. The white bar represents 10 μm. (**B**) The effect of decursin on the number of neurites in F11 cells. (**C**) The effect of decursin on the length of F11 neurites. NC: Negative control, treated with DMSO only. The number and length of F11 neurites shown in the graphs were determined from 10 images in each case, which were averaged. The number and length of F11 cells were normalized according to the F11 cells treated by DMSO only (NC), because the number and length of F11 cells treated by paclitaxel were extremely reduced, as seen in the middle panel of (A). * *p* < 0.05 and ** *p* < 0.01, determined using Student’s *t*-tests. This experiment was conducted in triplicate.

As stated earlier, patients with CINP and rodent models of CINP exhibit structural damage to the peripheral nervous system; thus, one potential strategy for treating CINP is facilitating the recovery of the damaged neuronal networks [1]. To test this, we exposed F11 cells in the presence of 10 nM of paclitaxel and treated cells with different concentrations of decursin. Thereafter, we assessed neurite outgrowth to determine the efficacy of decursin in facilitating neuronal differentiation and, by extension, recovery of the damaged neuronal network. Neurite number and length increased in a concentration-dependent manner following decursin treatment (Figure 2). Worth noting is that 2 μM of decursin increased the number and length of neurites by 2.41 times and 1.32 times, respectively (Figure 2B,C). This indicates that decursin can facilitate the regeneration of damaged neuronal systems.

### 3.3. Decursin Alleviated Mechanical Allodynia in the Mouse Model of Paclitaxel-Induced Peripheral Neuropathy

**Figure 3:**
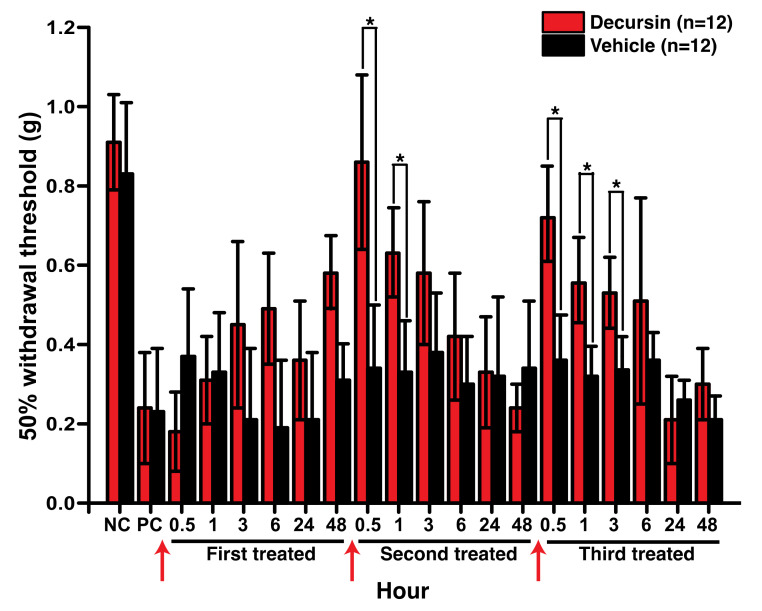
Effect of decursin on paclitaxel-induced neuropathic pain in mice. The mechanical allodynia test was performed immediately after injection of the vehicle and decursin. NC and PC indicate the negative control and paclitaxel-induced neuropathic pain mice, respectively. Red arrows indicate the time of decursin treatment. During the 1st, 2nd, and 3rd treatments, 50 mg/kg of decursin was administered via intrathecal injection. *n* indicates the number of mice in each group. * *p* < 0.05, determined using paired *t*-tests.

From the various taxane-derived anticancer drugs, we chose to use paclitaxel to develop the CINP model, via intraperitoneal injection. The level of mechanical allodynia was measured using the up-down method and von Frey filaments. First, we confirmed the establishment of the paclitaxel-induced model of neuropathic pain in experimental mice using the 50% withdrawal threshold before treatment with decursin; mice that exhibited a 50% withdrawal threshold were considerably more sensitive to mechanical allodynia than mice in the control group (Figure 3). The reduction of mechanical allodynia was not obvious after the first intrathecal injection of 50 mg/kg decursin (Figure 3). However, mechanical allodynia significantly decreased after the second injection of 50 mg/kg decursin; this decrease peaked immediately after the decursin injection and reduced over time (Figure 3). Interestingly, the reduction of mechanical allodynia, as a measure of the analgesic ability of decursin, was even more prolonged after the third injection of 50 mg/kg decursin (Figure 3). This demonstrated the analgesic ability of decursin in the in vivo model of paclitaxel-induced peripheral neuropathy.

### 3.4. Decursin Modulated Intracellular Ca^2+^ via TRPV1 Inhibition

**Figure 4:**
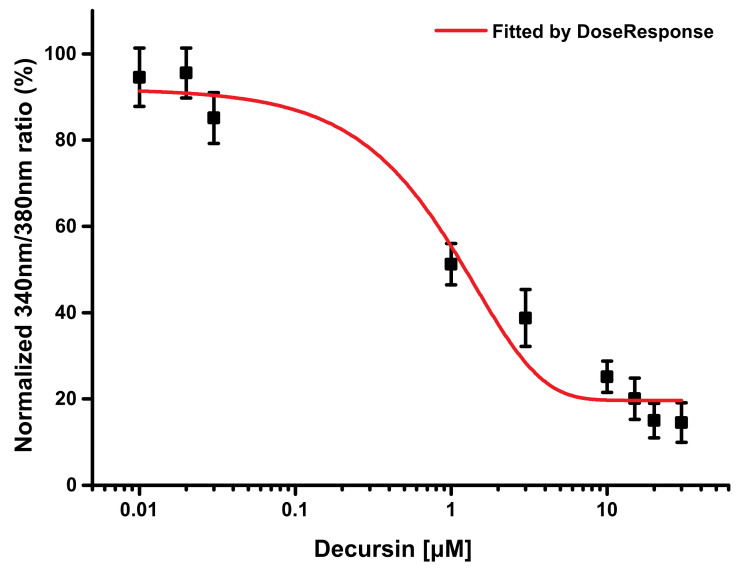
Intracellular Ca^2+^ levels after treatment with decursin in TRPV1-expressing HEK293 cells sensitized by capsaicin. These cells were exposed to different concentrations of decursin in the presence of capsaicin. The Δ340/380 ratio (%) at each decursin concentration was calculated by the same method as that reported in Figure 1B. The red line indicates the dose–response curve that was obtained using OriginPro software. This experiment was conducted in triplicate.

The sodium-channel protein type IX α subunit (SCN9A), TRPV1, and sodium-channel protein type X α subunit, which are classified as voltage-gated ion channels, were among the 100 protein targets predicted by the SwissTargetPrediction web tool (Table S1). Finally, we selected TRPV1 as a potential protein target because decursin reduced intracellular Ca^2+^ levels in the presence of capsaicin, and capsaicin is a known ligand of TRPV1 (Figure 1). When introduced to the human TRPV1 stable HEK293 cell line, decursin inhibited capsaicin-induced intracellular Ca^2+^ transients in a concentration-dependent manner (Figure 4). The IC_50_ of decursin against TRPV1 was 0.69 ± 0.105 μM, which is comparable to the 562 nM as IC_50_ of capsazepine, a specific TRPV1 antagonist [14]. This suggests that decursin acts as a selective TRPV1 inhibitor that modulates pain transmission that is mediated by the sudden increase in intracellular Ca^2+^.

### 3.5. Decursin Can Phosphorylate Protein Kinase B and cAMP-Response Element-Binding Protein, Related to Neurite Outgrowth

**Figure 5:**
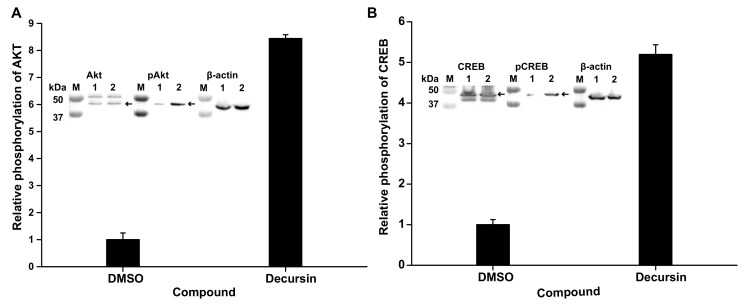
The phosphorylation of Atk and CREB following decursin treatment. (**A**) The phosphorylation of Akt in the presence of 2 μM decursin. (**B**) The phosphorylation of CREB in the presence of 2 μM decursin. Inlet pictures indicate immunoblotting results for Akt, phosphorylated Akt (pAkt), CREB, pCREB, and β-actin as a house-keeping protein. M, 1, and M2 indicate the protein size marker, the cell lysate without, and with decursin, respectively.

To identify how decursin can induce neurite outgrowth, we carried out immunoblotting against phosphorylated protein kinase B (Akt) and cAMP-response element-binding protein (CREB), which are related to neurite outgrowth, neuronal survival, dendritic growth, and changes in spine morphology [15] in the presence of decursin. In the presence of 2 μM decursin, the phosphorylation level of Akt and CREB increased by 8.44- and 5.19-fold, respectively (Figure 5). This result shows that decursin induced neurite outgrowth of F11 cells in the presence of paclitaxel (Figure 2 and Figure 5).

## 4. Discussion

CINP is a severe side-effect experienced by patients undergoing treatment with platinum- and taxane-derived anticancer drugs, such as oxaliplatin, cisplatin, carboplatin, and paclitaxel [1]. CINP is often severe, including paresthesia and dysesthesia; this severe pain can indirectly reduce the efficacy of anticancer drugs, as it may be detrimental to patients’ will to fight for survival [2,3]. Paclitaxel-induced peripheral neuropathy is characterized by neuronal network damage, such as axonal degradation and loss of intraepidermal nerve fibers, which are caused by oxidative stress, altered microtubular dynamics, mitochondrial dysfunction, and inflammation [16,17]. Moreover, TRPs are known to participate in pain transition by effecting a sudden elevation in Ca^2+^ levels that can lead to inflammation, tissue remodeling, and neuronal plasticity, in CINP and various types of chronic pain [18]. Therefore, therapeutic agents to treat CINP could include drugs that facilitate the effective repair of damaged neuronal networks and inhibit the pain transition that is caused by the sudden increase in Ca^2+^, which would ultimately alleviate pain.

Decursin has been reported to have various biological effects, such as anti-inflammatory activity, anticancer activity, blood circulation enhancement, and wound healing [6]. In particular, the anti-inflammatory and cellular remodeling functions of decursin in wound healing can promote the recovery of damage to the peripheral nervous system [7]. The analgesic effect of decursin on mechanical allodynia in the mice with paclitaxel-induced peripheral neuropathy was prolonged and persisted longer after repeated treatment in this study. Corresponding to this, a pharmacokinetic study for decursin in rats indicated that the peak plasma concentration was 43.7 ± 57.6 ng/mL and the time for the peak plasma concentration was 0.5 ± 0 h when orally administered at a single dose of 50 mg/kg [19]. Moreover, decursinol, the hydrolysis product of decursin, was detectable as soon as 5 min (9.3 μg/mL) after intraperitoneal injection of 4.8 mg decursin in mice, and its level went up to 79.7 μg/mL at 3 h [20].

We also examined the in vitro effects of decursin as related to neurite outgrowth resulting alleviation of paclitaxel-induced peripheral neuropathy in this study. The analysis of neurite outgrowth showed that decursin dramatically improved the number and length of neurites in the presence of paclitaxel, and increased the phosphorylation of Akt and CREB proteins, as revealed by immunoblotting. Previous studies have reported that the reduction of Akt and CREB phosphorylation can cause neuron damage and loss in animal models of neurodegenerative disease [21,22,23,24]. However, enhancing Akt/CREB activity via phosphorylation can contribute to inhibition of neuroinflammation and improvements in neuronal function, bringing benefits such as neurite outgrowth, neuronal survival, dendritic growth, and changes in spine morphology [15]. Previous studies have also investigated the analgesic potential of ghrelin and phosphatidylcholine for alleviating paclitaxel-induced peripheral neuropathy; interestingly, these compounds were found to directly repair the neuronal network damaged by paclitaxel injection in mouse models [25,26]. Therefore, the decursin-induced improvement in the number and length of neurites, directly and indirectly, influenced the recovery of neuronal networks damaged by axonal degradation and loss of intraepidermal nerve fibers. We found that decursin reduced intracellular Ca^2+^ levels in the presence of capsaicin via TRPV1 inhibition. As mentioned above, TRPV1 is an interesting target protein for treating CINP, because it directly mediates pain transition via a sudden increase in intracellular Ca^2+^. Previous in vivo studies have reported that AMG9801 and NEO6860 improved paclitaxel-induced peripheral neuropathy by inhibiting the function of TRPV1 [27,28]. Previous work has also shown that the expression of TRPV1 in small dorsal root ganglion neurons of rats was increased by treatment of oxaliplatin, which is an agent used in chemotherapy [27,28]. This increased TRPV1 expression contributed to the development of mechanical allodynia and thermal hyperalgesia in oxaliplatin-induced peripheral neuropathic pain rats [29,30]. Therefore, these results show that controlling TRPV1 activity is one strategy to treat CINP.

These results imply that decursin could act as an analgesic by modulating the dysregulated signal transduction caused by sudden Ca^2+^ elevation and damage to neuronal networks, which are typically observed in models of peripheral neuropathic pain. Thus, we investigated the effect of decursin in paclitaxel-induced peripheral neuropathic mice. We observed a considerable reduction in mechanical allodynia, and this effect was enhanced by an increase in the frequency of decursin treatment. Therefore, we concluded that decursin could be a promising candidate for the treatment of CINP owing to its dual and simultaneous mechanism of action, i.e., the recovery of damaged neuronal networks and TRPV1 inhibition.

## Data Availability

The data presented in this study are available in the article or Supplementary Materials.

## Supplementary Materials

The following are available online at https://www.mdpi.com/2073-4409/10/3/547/s1: Figure S1: Intracellular Ca_2+_ levels following decursin treatment in F11 cells in the presence of menthol, Table S1: List of protein targets of decursin detected by the SwissTargetPrediction web tool.
