# Supplementary material for: Decursin Alleviates Mechanical Allodynia in a Paclitaxel-Induced Neuropathic Pain Mouse Model
Source: Cells. 2021 Mar 4;10(3):547. doi: 10.3390/cells10030547 (PMC8001788; doi:10.3390/cells10030547)
Supplement: Supplementary file 1 [file cells-10-00547-s001.pdf]

cells

MDPI

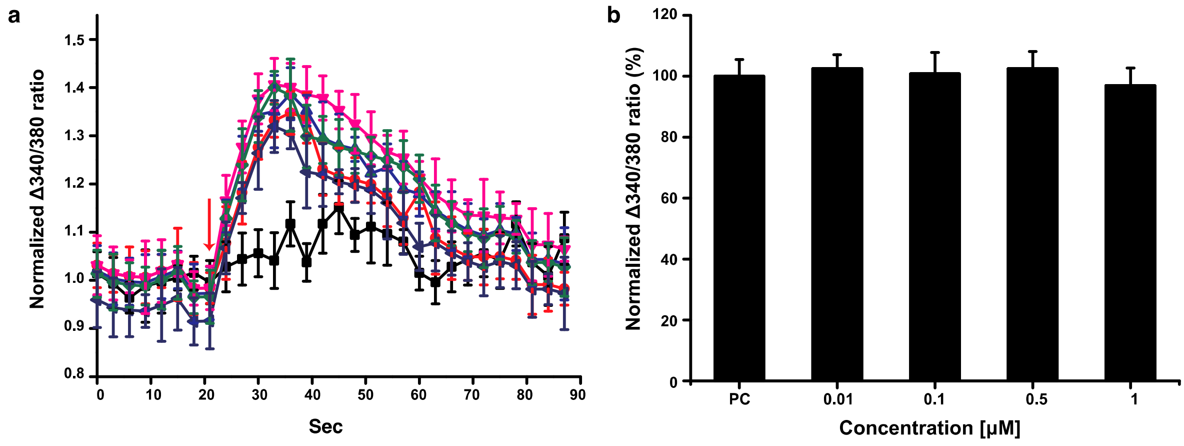

Supplementary Figure S1. Intracellular Ca^{2+} levels following decursin treatment in F11 cells in the presence of menthol

(A) Intracellular Ca^{2+} levels after treatment with decursin every 3 s. The red arrow indicates the duration of treatment with 200 μM of menthol and decursin. NC: The negative control treated with DMSO only; PC: The positive control treated with 200 μM menthol without decursin. (B) Average intracellular Ca^{2+} levels after treatment with decursin. The average was quantified from the normalized Δ340/380 ratio for 10 cycles after treatment with the decursin solution at the 10th cycle, as shown in Fig. 1A. The normalized Δ340/380 ratio was calculated using the following formula: [ratio of fluorescence intensity at 510 nm (emission) to that at 340 nm (excitation)]/[ratio of fluorescence intensity at 510 nm (emission) to that at a wavelength of 380 nm (excitation)].

Table S1. List of protein targets of decursin detected by the SwissTargetPrediction web tool

Target	Common name	Uniprot ID	ChEMBL ID	Target Class	Probability
Poly [ADP-ribose] polymerase-1	PARP1	P09874	CHEMBL3105	Enzyme	0.104671941
N-acylsphingosine-amidohydrolase	NAAA	Q02083	CHEMBL4349	Enzyme	0.104671941
Acid ceramidase	ASAH1	Q13510	CHEMBL5463	Enzyme	0.104671941
Neuropeptide Y receptor type 5	NPY5R	Q15761	CHEMBL4561	Family A G protein-coupled receptor	0.104671941
Melatonin receptor 1A	MTNR1A	P48039	CHEMBL1945	Family A G protein-coupled receptor	0.104671941
Melatonin receptor 1B	MTNR1B	P49286	CHEMBL1946	Family A G protein-coupled receptor	0.104671941
Phosphodiesterase 4D	PDE4D	Q08499	CHEMBL288	Phosphodiesterase	0.104671941
Glucagon receptor	GCGR	P47871	CHEMBL1985	Family B G protein-coupled receptor	0.104671941
Corticotropin releasing factor receptor 1	CRHR1	P34998	CHEMBL1800	Family B G protein-coupled receptor	0.104671941
Cathepsin K	CTSK	P43235	CHEMBL268	Protease	0.104671941
Metabotropic glutamate receptor 5	GRM5	P41594	CHEMBL3227	Family C G protein-coupled receptor	0.104671941
Metabotropic glutamate receptor 1	GRM1	Q13255	CHEMBL3772	Family C G protein-coupled receptor	0.104671941
Isocitrate dehydrogenase (NADP) cytoplasmic	IDH1	O75874	CHEMBL2007625	Enzyme	0.104671941
Serine/threonine-protein kinase Aurora-B	AURKB	Q96GD4	CHEMBL2185	Kinase	0.104671941
Glycogen synthase kinase-3 beta	GSK3B	P49841	CHEMBL262	Kinase	0.104671941
Tyrosine-protein kinase JAK2	JAK2	O60674	CHEMBL2971	Kinase	0.104671941
Rho-associated protein kinase 2	ROCK2	O75116	CHEMBL2973	Kinase	0.104671941
UDP-N-acetylglucosamine--peptide N-Acetylglucosaminyltransferase 110 kDa subunit	OGT	O15294	CHEMBL5955	Enzyme	0.104671941
Ribosomal protein S6 kinase 1	RPS6KB1	P23443	CHEMBL4501	Kinase	0.104671941
Serine/threonine-protein kinase Aurora-A	AURKA	O14965	CHEMBL4722	Kinase	0.104671941
Acyl-CoA desaturase (by homology)	SCD	O00767	CHEMBL5555	Enzyme	0.104671941
Tyrosine-protein kinase SRC	SRC	P12931	CHEMBL267	Kinase	0.104671941
Sodium channel protein type IX alpha subunit	SCN9A	Q15858	CHEMBL4296	Voltage-gated ion channel	0.104671941
dUTP pyrophosphatase	DUT	P33316	CHEMBL5203	Enzyme	0.104671941
Tyrosine-protein kinase ABL	ABL1	P00519	CHEMBL1862	Kinase	0.104671941
Cyclin-dependent kinase 1/cyclin B	CCNB3 CDK1	Q8WWL7 P06493	CHEMBL2094127	Other cytosolic protein	0.104671941

	CCNB1<br>CCNB2	P14635<br>O95067			
Serine/threonine-protein kinase PIM1	PIM1	P11309	CHEMBL2147	Kinase	0.104671941
Phosphodiesterase 4A	PDE4A	P27815	CHEMBL254	Phosphodiesterase	0.104671941
Phosphodiesterase 4B	PDE4B	Q07343	CHEMBL275	Phosphodiesterase	0.104671941
Phosphodiesterase 4C	PDE4C	Q08493	CHEMBL291	Phosphodiesterase	0.104671941
Vanilloid receptor	TRPV1	Q8NER1	CHEMBL4794	Voltage-gated ion channel	0.104671941
Serotonin 1a (5-HT1a) receptor	HTR1A	P08908	CHEMBL214	Family A G protein-coupled receptor	0.104671941
Protein-tyrosine phosphatase 1B	PTPN1	P18031	CHEMBL335	Phosphatase	0.104671941
Matrix metalloproteinase 1	MMP1	P03956	CHEMBL332	Protease	0.104671941
Mitogen-activated protein kinase kinase kinase kinase 4	MAP4K4	O95819	CHEMBL6166	Kinase	0.104671941
15-hydroxyprostaglandin dehydrogenase [NAD+]	HPGD	P15428	CHEMBL1293255	Enzyme	0.104671941
Dopamine D1 receptor	DRD1	P21728	CHEMBL2056	Family A G protein-coupled receptor	0.104671941
Free fatty acid receptor 1	FFAR1	O14842	CHEMBL4422	Family A G protein-coupled receptor	0.104671941
Orexin receptor 2	HCRTR2	O43614	CHEMBL4792	Family A G protein-coupled receptor	0.104671941
Orexin receptor 1	HCRTR1	O43613	CHEMBL5113	Family A G protein-coupled receptor	0.104671941
Cyclin-dependent kinase 2/cyclin A	CDK2<br>CCNA1<br>CCNA2	P24941<br>P78396<br>P20248	CHEMBL2094128	Other cytosolic protein	0.104671941
Phosphodiesterase 3	PDE3A	Q14432	CHEMBL241	Phosphodiesterase	0.104671941
Phosphodiesterase 3B	PDE3B	Q13370	CHEMBL290	Phosphodiesterase	0.104671941
Serine/threonine-protein kinase PLK1	PLK1	P53350	CHEMBL3024	Kinase	0.104671941
Adenosine kinase	ADK	P55263	CHEMBL3589	Enzyme	0.104671941
11-beta-hydroxysteroid dehydrogenase 1	HSD11B1	P28845	CHEMBL4235	Enzyme	0.104671941
Serine/threonine-protein kinase PLK3	PLK3	Q9H4B4	CHEMBL4897	Kinase	0.104671941
Sodium/glucose cotransporter 1	SLC5A1	P13866	CHEMBL4979	Electrochemical transporter	0.104671941
G protein-coupled receptor kinase 5	GRK5	P34947	CHEMBL5678	Kinase	0.104671941
Serine/threonine-protein kinase PLK2	PLK2	Q9NYY3	CHEMBL5938	Kinase	0.104671941
Epoxide hydratase	EPHX2	P34913	CHEMBL2409	Protease	0.104671941
PI3-kinase p110-gamma subunit	PIK3CG	P48736	CHEMBL3267	Enzyme	0.104671941

Metabotropic glutamate receptor 2	GRM2	Q14416	CHEMBL5137	Family C G protein-coupled receptor	0.104671941
Bromodomain-containing protein 4	BRD4	O60885	CHEMBL1163125	Reader	0.104671941
Serine/threonine-protein kinase Chk2	CHEK2	O96017	CHEMBL2527	Kinase	0.104671941
Tyrosine-protein kinase JAK1	JAK1	P23458	CHEMBL2835	Kinase	0.104671941
Phosphodiesterase 10A	PDE10A	Q9Y233	CHEMBL4409	Phosphodiesterase	0.104671941
Alpha-synuclein	SNCA	P37840	CHEMBL6152	Unclassified protein	0.104671941
Muscarinic acetylcholine receptor M1 (by homology)	CHRM1	P11229	CHEMBL216	Family A G protein-coupled receptor	0.104671941
Vascular endothelial growth factor receptor 2	KDR	P35968	CHEMBL279	Kinase	0.104671941
5-lipoxygenase activating protein	ALOX5AP	P20292	CHEMBL4550	Other cytosolic protein	0.104671941
Cathepsin S	CTSS	P25774	CHEMBL2954	Protease	0.104671941
Cathepsin (B and K)	CTSB	P07858	CHEMBL4072	Protease	0.104671941
Pyruvate kinase isozymes M1/M2	PKM	P14618	CHEMBL1075189	Enzyme	0.104671941
CDK8/Cyclin C	CCNC CDK8	P24863 P49336	CHEMBL3038474	Kinase	0.104671941
CDC7/DBF4 (Cell division cycle 7-related protein kinase/Activator of S phase kinase)	CDC7	O00311	CHEMBL5443	Kinase	0.104671941
Cell division protein kinase 8	CDK8	P49336	CHEMBL5719	Kinase	0.104671941
Steroid 5-alpha-reductase 2	SRD5A2	P31213	CHEMBL1856	Oxidoreductase	0.104671941
Protein farnesyltransferase	FNTA FNTB	P49354 P49356	CHEMBL2094108	Enzyme	0.104671941
ADAMTS5	ADAMTS5	Q9UNA0	CHEMBL2285	Protease	0.104671941
Poly [ADP-ribose] polymerase 2	PARP2	Q9UGN5	CHEMBL5366	Enzyme	0.104671941
Complement factor D	CFD	P00746	CHEMBL2176771	Protease	0.104671941
Thromboxane-A synthase	TBXAS1	P24557	CHEMBL1835	Cytochrome P450	0.104671941
Serine/threonine-protein kinase RAF	RAF1	P04049	CHEMBL1906	Kinase	0.104671941
Tyrosyl-DNA phosphodiesterase 2	TDP2	O95551	CHEMBL2169736	Enzyme	0.104671941
Intercellular adhesion molecule-1	ICAM1	P05362	CHEMBL3070	Adhesion	0.104671941
Vascular cell adhesion protein 1	VCAM1	P19320	CHEMBL3735	Adhesion	0.104671941
Selectin E	SELE	P16581	CHEMBL3890	Adhesion	0.104671941
Nuclear receptor ROR-gamma	RORC	P51449	CHEMBL1741186	Nuclear receptor	0.104671941
Matrix metalloproteinase 13	MMP13	P45452	CHEMBL280	Protease	0.104671941
Matrix metalloproteinase 3	MMP3	P08254	CHEMBL283	Protease	0.104671941
Cyclin-dependent kinase 2	CDK2	P24941	CHEMBL301	Kinase	0.104671941
Matrix metalloproteinase 9	MMP9	P14780	CHEMBL321	Protease	0.104671941
Matrix metalloproteinase 8	MMP8	P22894	CHEMBL4588	Protease	0.104671941
Neuronal acetylcholine receptor; alpha4/beta2	CHRNA4 CHRNB2	P43681 P17787	CHEMBL1907589	Ligand-gated ion channel	0.104671941

Cyclin-dependent kinase 4/cyclin D1	CCND1<br>CDK4	P24385<br>P11802	CHEMBL1907601	Kinase	0.104671941
Dopamine D4 receptor	DRD4	P21917	CHEMBL219	Family A G protein-coupled receptor	0.104671941
Dopamine D3 receptor	DRD3	P35462	CHEMBL234	Family A G protein-coupled receptor	0.104671941
Neuronal acetylcholine receptor protein alpha-7 subunit	CHRNA7	P36544	CHEMBL2492	Ligand-gated ion channel	0.104671941
Estradiol 17-beta-dehydrogenase 2	HSD17B2	P37059	CHEMBL2789	Enzyme	0.104671941
Estradiol 17-beta-dehydrogenase 1	HSD17B1	P14061	CHEMBL3181	Enzyme	0.104671941
Urokinase-type plasminogen activator	PLAU	P00749	CHEMBL3286	Protease	0.104671941
NAD-dependent deacetylase sirtuin 2	SIRT2	Q8IXJ6	CHEMBL4462	Eraser	0.104671941
Serine/threonine-protein kinase PIM2	PIM2	Q9P1W9	CHEMBL4523	Kinase	0.104671941
Sodium channel protein type X alpha subunit (by homology)	SCN10A	Q9Y5Y9	CHEMBL5451	Voltage-gated ion channel	0.104671941
Interferon-induced, double-stranded RNA-activated protein kinase	EIF2AK2	P19525	CHEMBL5785	Kinase	0.104671941
Glutamate receptor ionotropic, AMPA 1	GRIA1	P42261	CHEMBL2009	Ligand-gated ion channel	0.104671941
GABA-A receptor; alpha-3/beta-3/gamma-2	GABRB3<br>GABRA3<br>GABRG2	P28472<br>P34903<br>P18507	CHEMBL2094120	Ligand-gated ion channel	0.104671941
GABA-A receptor; alpha-1/beta-3/gamma-2	GABRB3<br>GABRG2<br>GABRA1	P28472<br>P18507<br>P14867	CHEMBL2094121	Ligand-gated ion channel	0.104671941
GABA-A receptor; alpha-5/beta-3/gamma-2	GABRB3<br>GABRG2<br>GABRA5	P28472<br>P18507<br>P31644	CHEMBL2094122	Ligand-gated ion channel	0.104671941
